# Supplementary material for: Nociception testing during fixed-wing ambulance flights. An interventional pilot study on the effects of flight-related environmental changes on the nociception of healthy volunteers
Source: PLoS One. 2020 Feb 24;15(2):e0217530. doi: 10.1371/journal.pone.0217530 (PMC7039504; doi:10.1371/journal.pone.0217530)
Supplement: S1 File — (PDF) [file pone.0217530.s001.pdf]

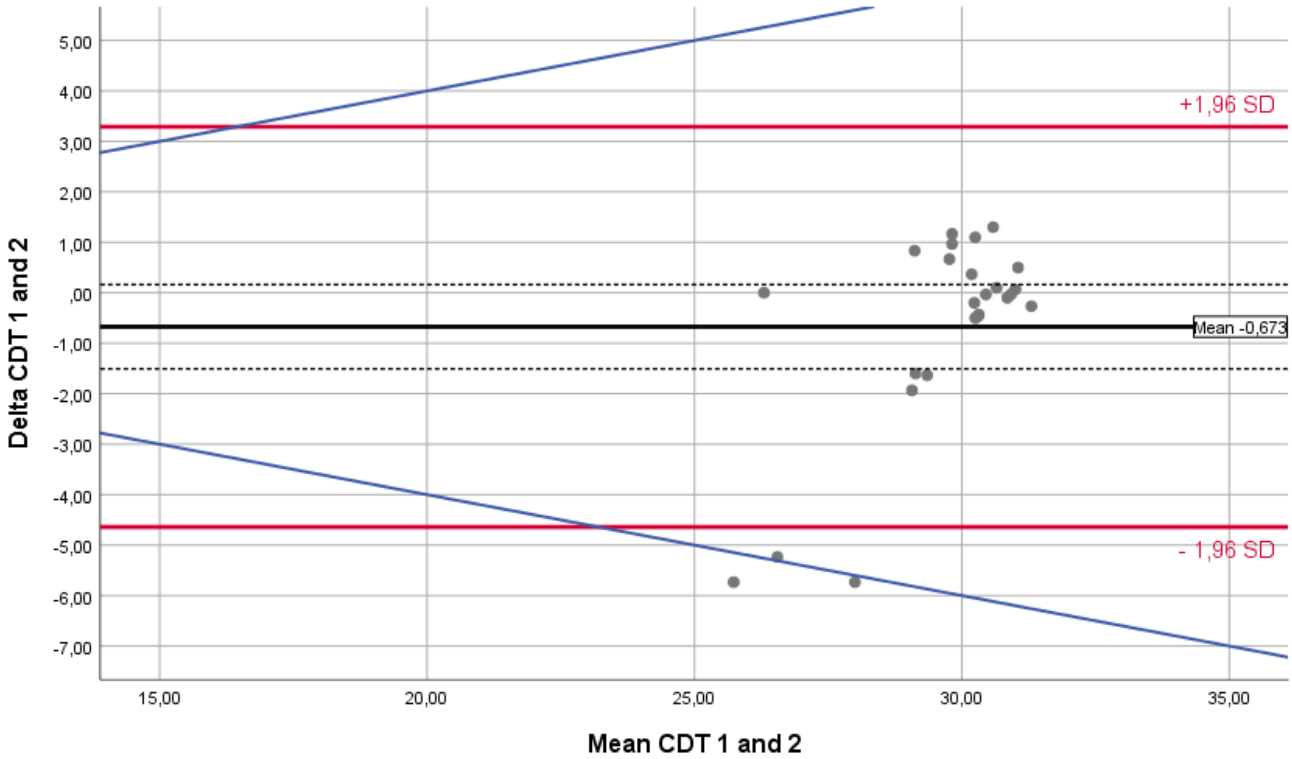

Supporting Information Figure 1:

Bland Altman Plot – Cold Detection Threshold (CDT) – Waypoints 1 against 2

The solid black lines in the Bland-Altman-Plots represent the mean of the differences.
The confidence intervals for the means of differences are depicted as dashed black lines.
The red upper (lower) lines show the upper (lower) limits of agreement equal to mean ± 1.96 SD.
The blue lines represent a margin of ±20% around the means of measurements from each modality and serve as a possible indicator of clinical relevance.

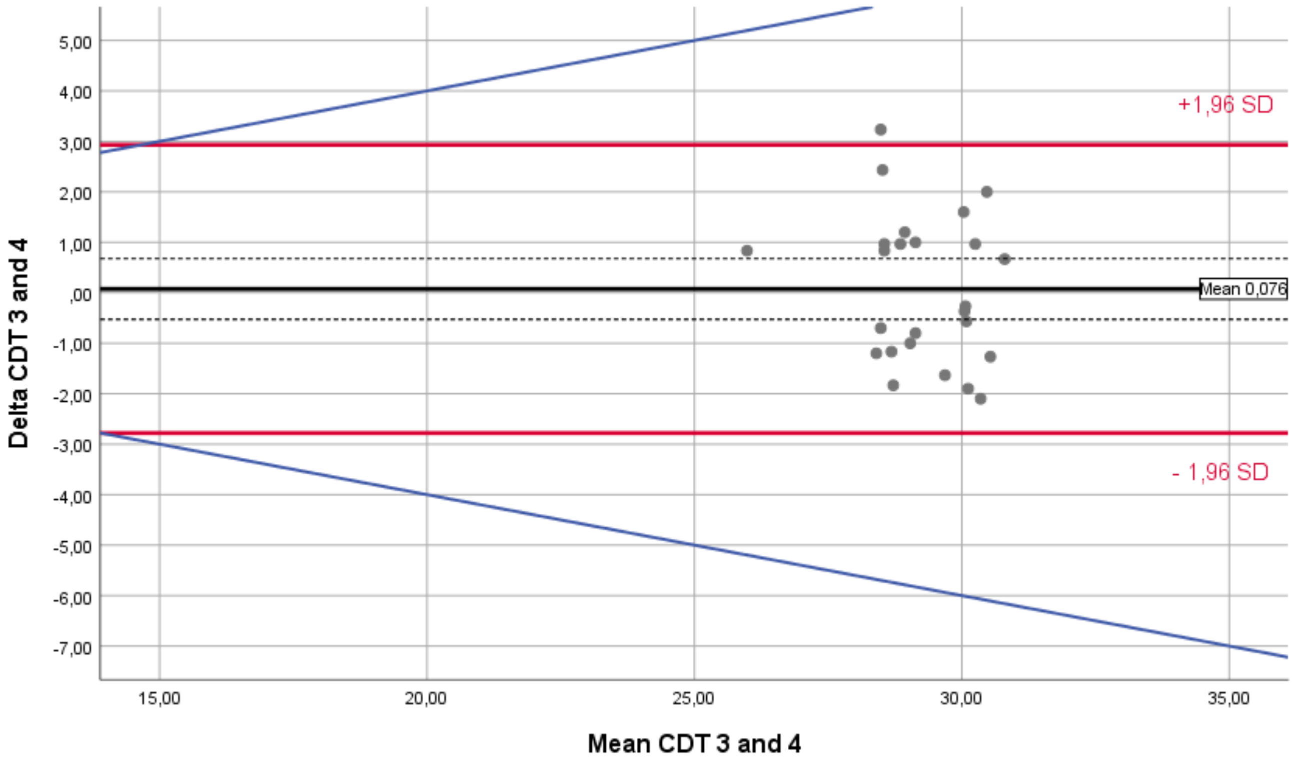

Supporting Information Figure 2:

Bland Altman Plot – Cold Detection Threshold (CDT) – Waypoints 3 against 4

The solid black lines in the Bland-Altman-Plots represent the mean of the differences.
The confidence intervals for the means of differences are depicted as dashed black lines.
The red upper (lower) lines show the upper (lower) limits of agreement equal to mean ± 1.96 SD.
The blue lines represent a margin of ±20% around the means of measurements from each modality and serve as a possible indicator of clinical relevance.

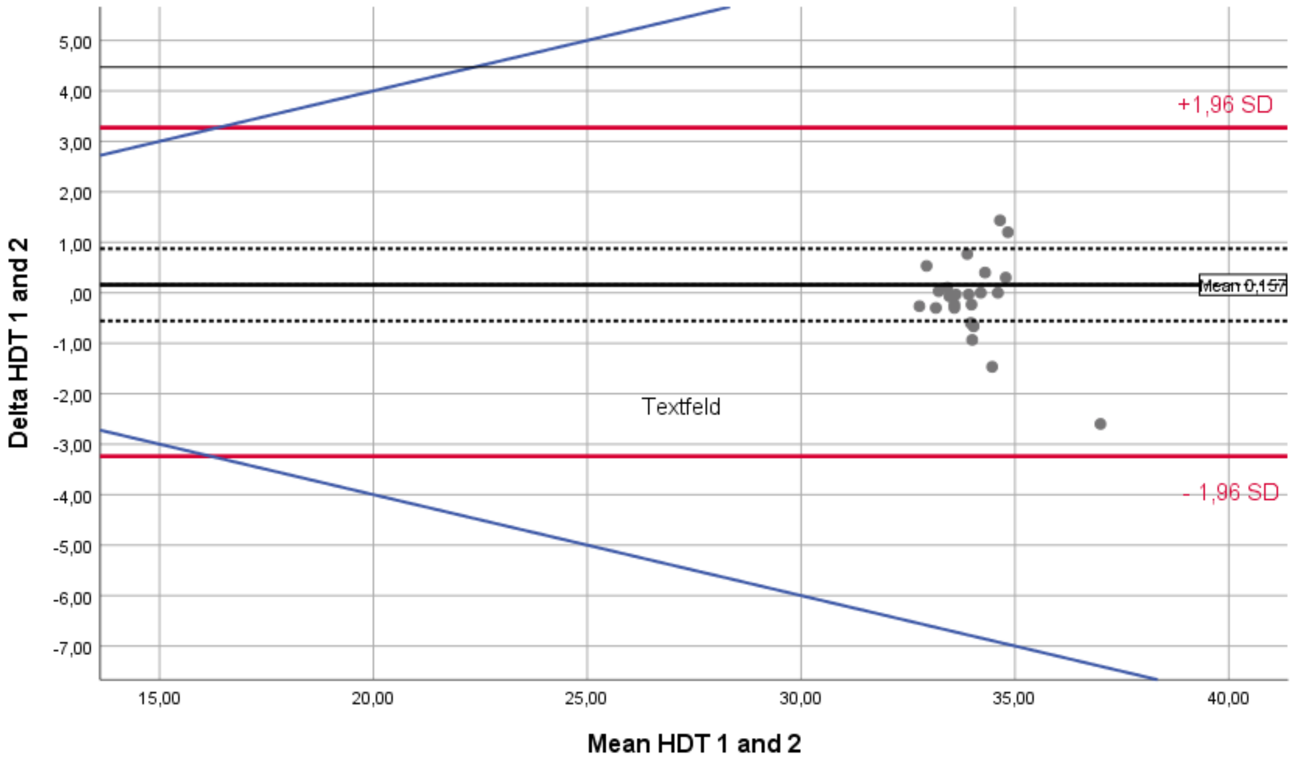

Supporting Information Figure 3:

Bland Altman Plot – Heat Detection Threshold (CDT) – Waypoints 1 against 2

The solid black lines in the Bland-Altman-Plots represent the mean of the differences.
The confidence intervals for the means of differences are depicted as dashed black lines.
The red upper (lower) lines show the upper (lower) limits of agreement equal to mean ± 1.96 SD.
The blue lines represent a margin of ±20% around the means of measurements from each modality and serve as a possible indicator of clinical relevance.

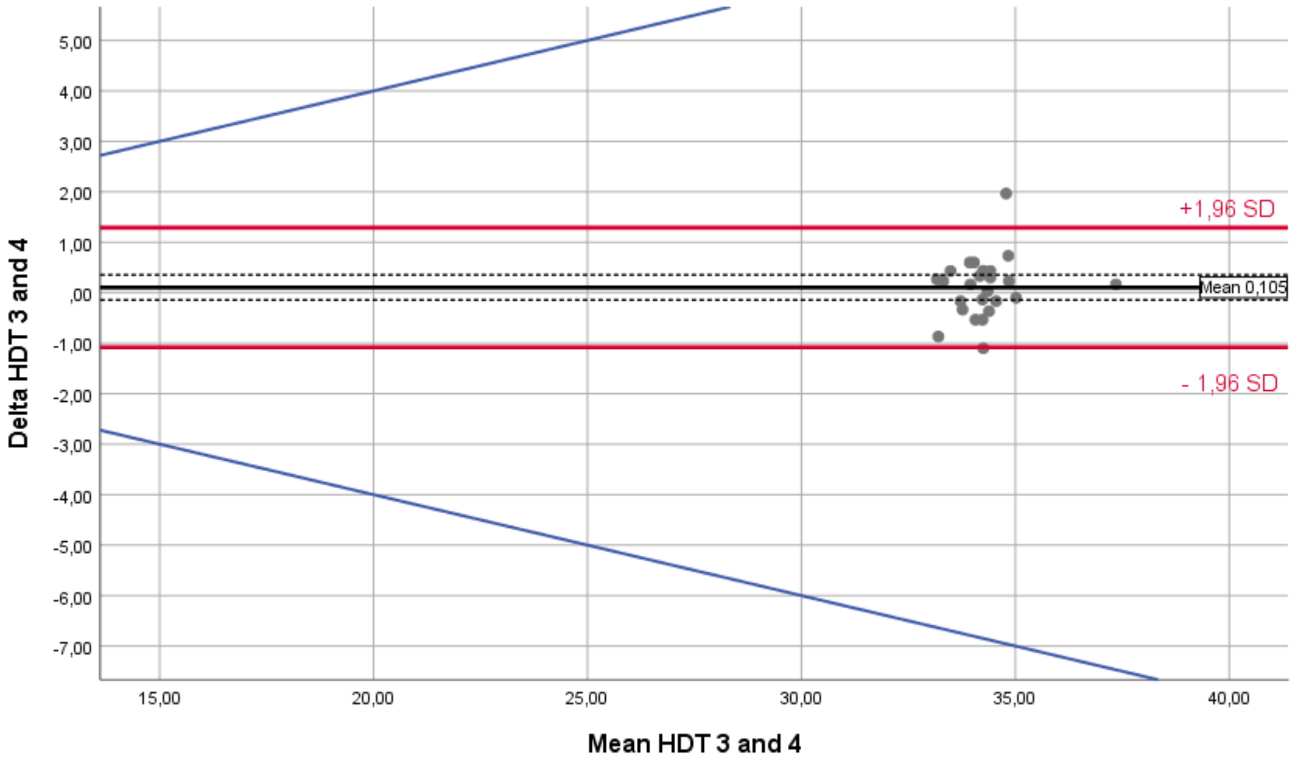

Supporting Information Figure 4:

Bland Altman Plot – Heat Detection Threshold (HDT) – Waypoints 3 against 4

The solid black lines in the Bland-Altman-Plots represent the mean of the differences.
The confidence intervals for the means of differences are depicted as dashed black lines.
The red upper (lower) lines show the upper (lower) limits of agreement equal to mean ± 1.96 SD.
The blue lines represent a margin of ±20% around the means of measurements from each modality and serve as a possible indicator of clinical relevance.

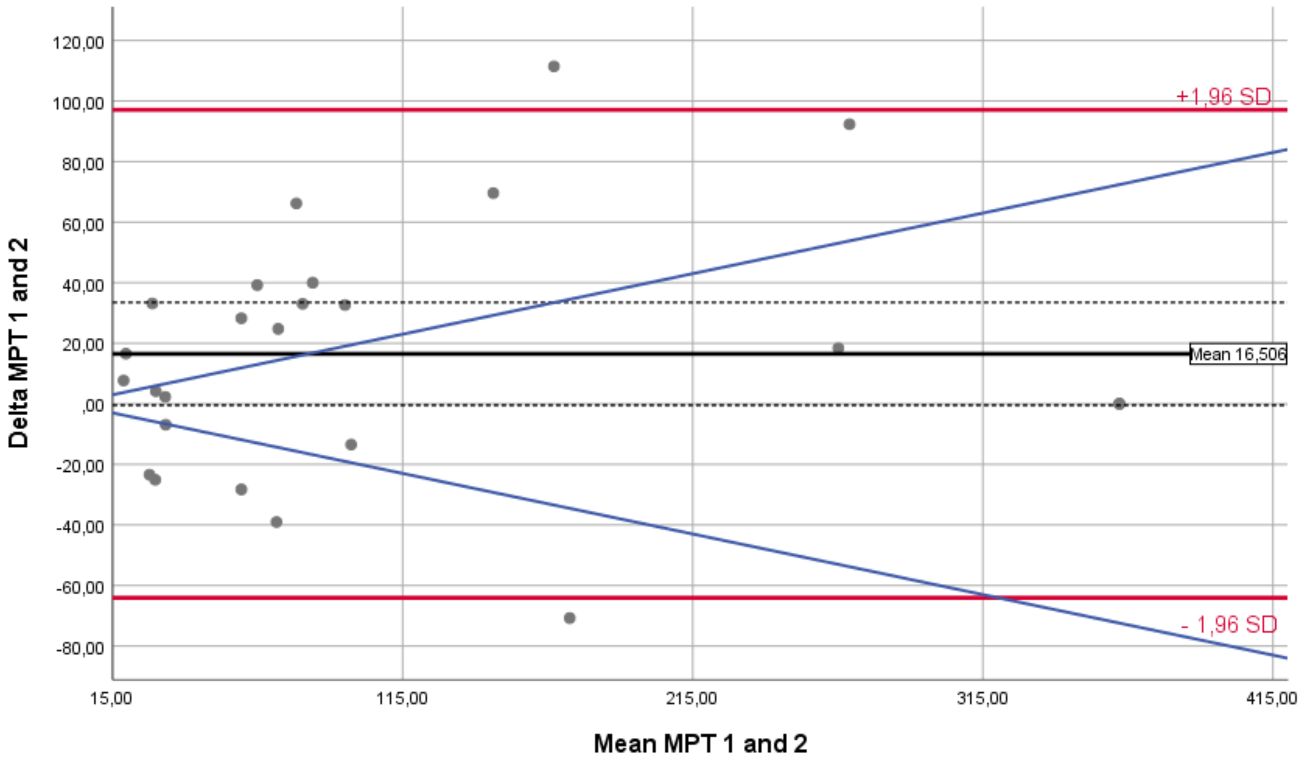

Supporting Information Figure 5:

Bland Altman Plot – Mechanical Pain Threshold (MPT) – Waypoints 1 against 2

The solid black lines in the Bland-Altman-Plots represent the mean of the differences.
The confidence intervals for the means of differences are depicted as dashed black lines.
The red upper (lower) lines show the upper (lower) limits of agreement equal to mean ± 1.96 SD.
The blue lines represent a margin of ±20% around the means of measurements from each modality and serve as a possible indicator of clinical relevance.

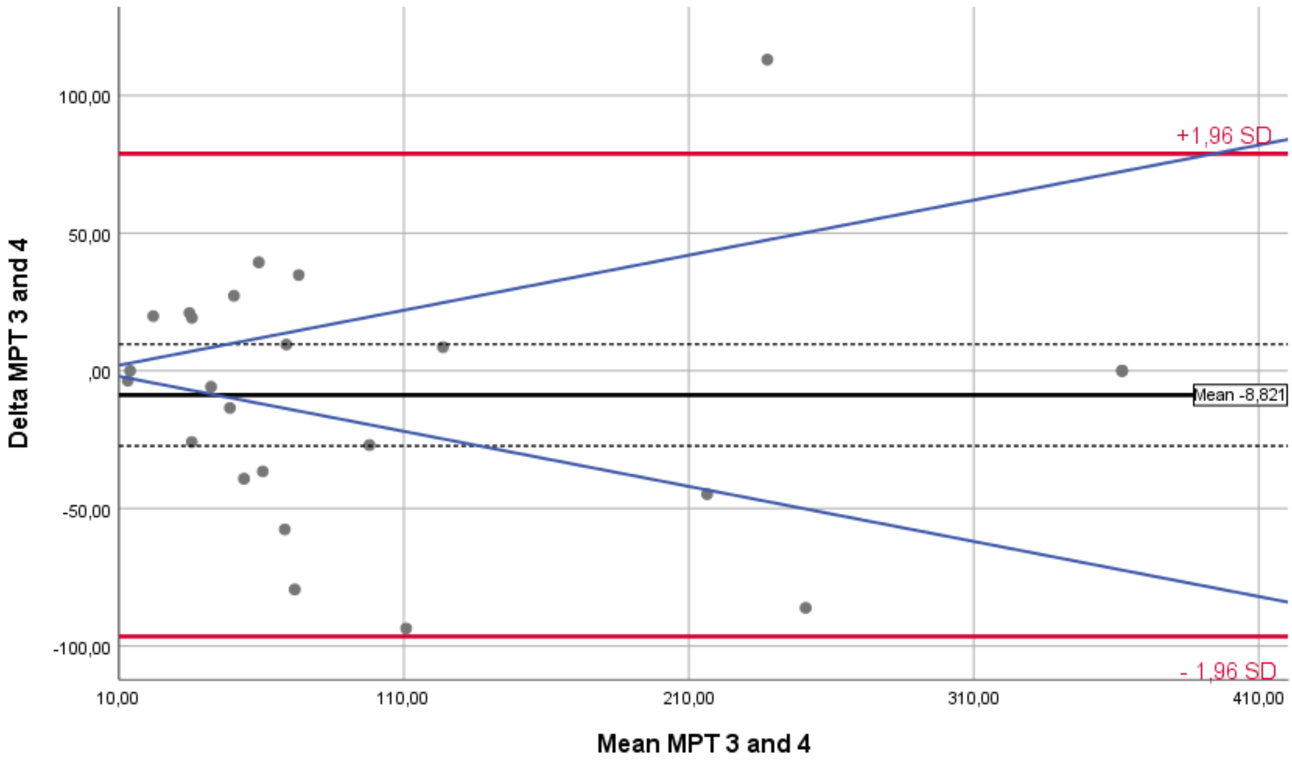

Supporting Information Figure 6:

Bland Altman Plot – Mechanical Pain Threshold (MPT) – Waypoints 3 against 4

The solid black lines in the Bland-Altman-Plots represent the mean of the differences.
The confidence intervals for the means of differences are depicted as dashed black lines.
The red upper (lower) lines show the upper (lower) limits of agreement equal to mean ± 1.96 SD.
The blue lines represent a margin of ±20% around the means of measurements from each modality and serve as a possible indicator of clinical relevance.

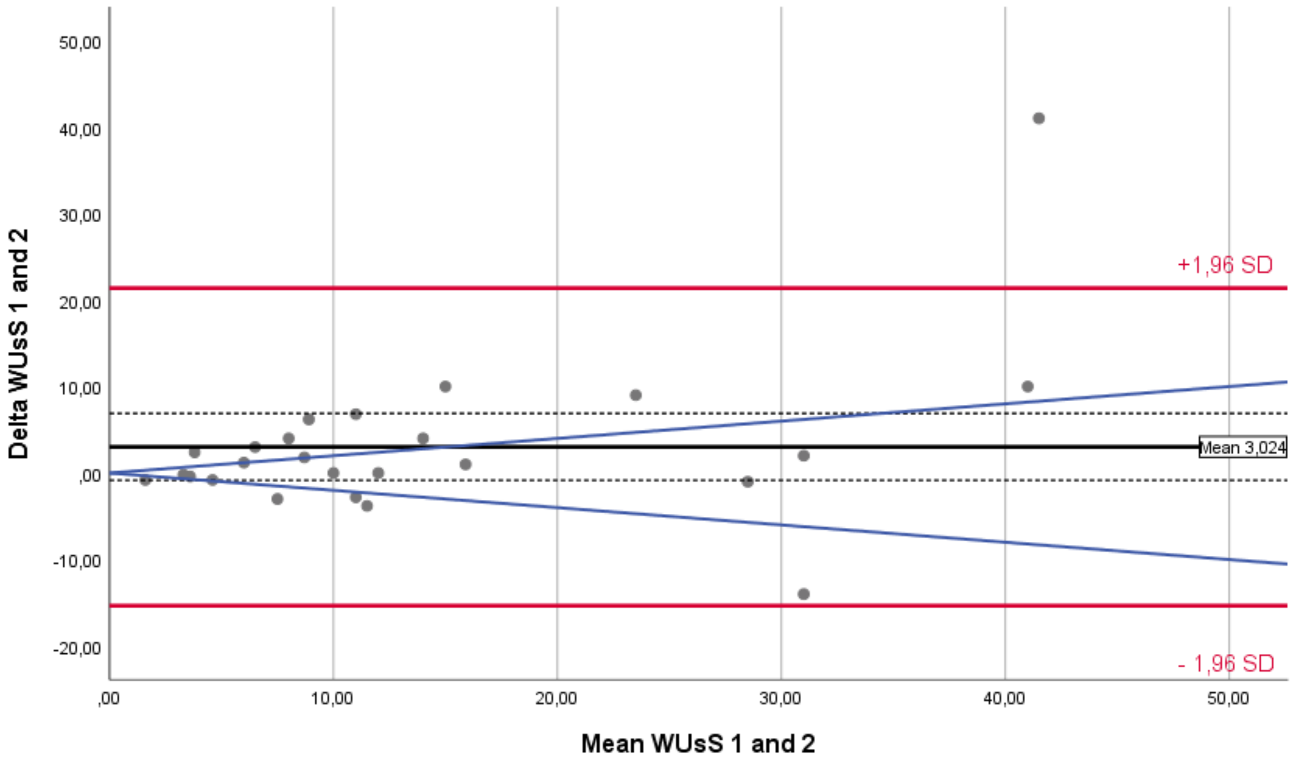

Supporting Information Figure 7:

Bland Altman Plot – Wind Up single Stimulus (WUsS) – Waypoints 1 against 2

The solid black lines in the Bland-Altman-Plots represent the mean of the differences.
The confidence intervals for the means of differences are depicted as dashed black lines.
The red upper (lower) lines show the upper (lower) limits of agreement equal to mean ± 1.96 SD.
The blue lines represent a margin of ±20% around the means of measurements from each modality and serve as a possible indicator of clinical relevance.

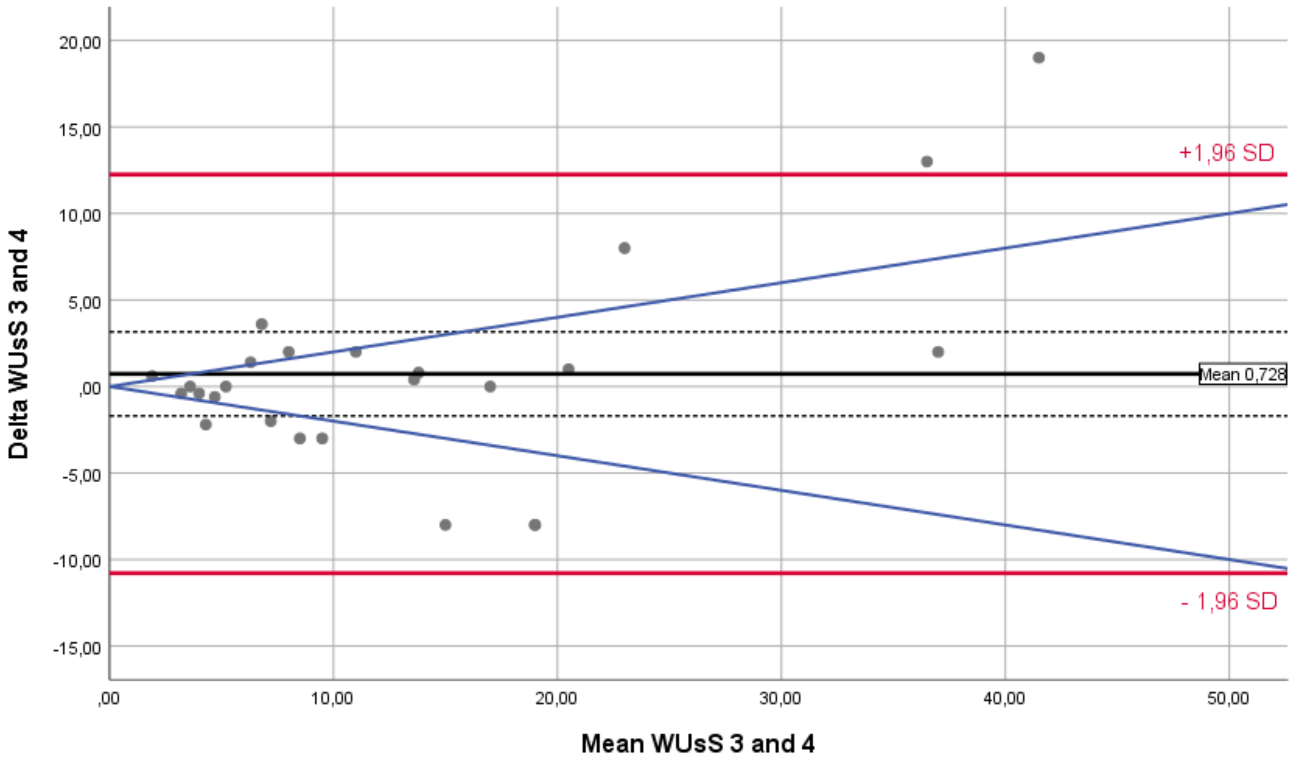

Supporting Information Figure 8:

Bland Altman Plot – Wind Up single stimulus (WUsS) – Waypoints 3 against 4

The solid black lines in the Bland-Altman-Plots represent the mean of the differences.
The confidence intervals for the means of differences are depicted as dashed black lines.
The red upper (lower) lines show the upper (lower) limits of agreement equal to mean ± 1.96 SD.
The blue lines represent a margin of ±20% around the means of measurements from each modality and serve as a possible indicator of clinical relevance.

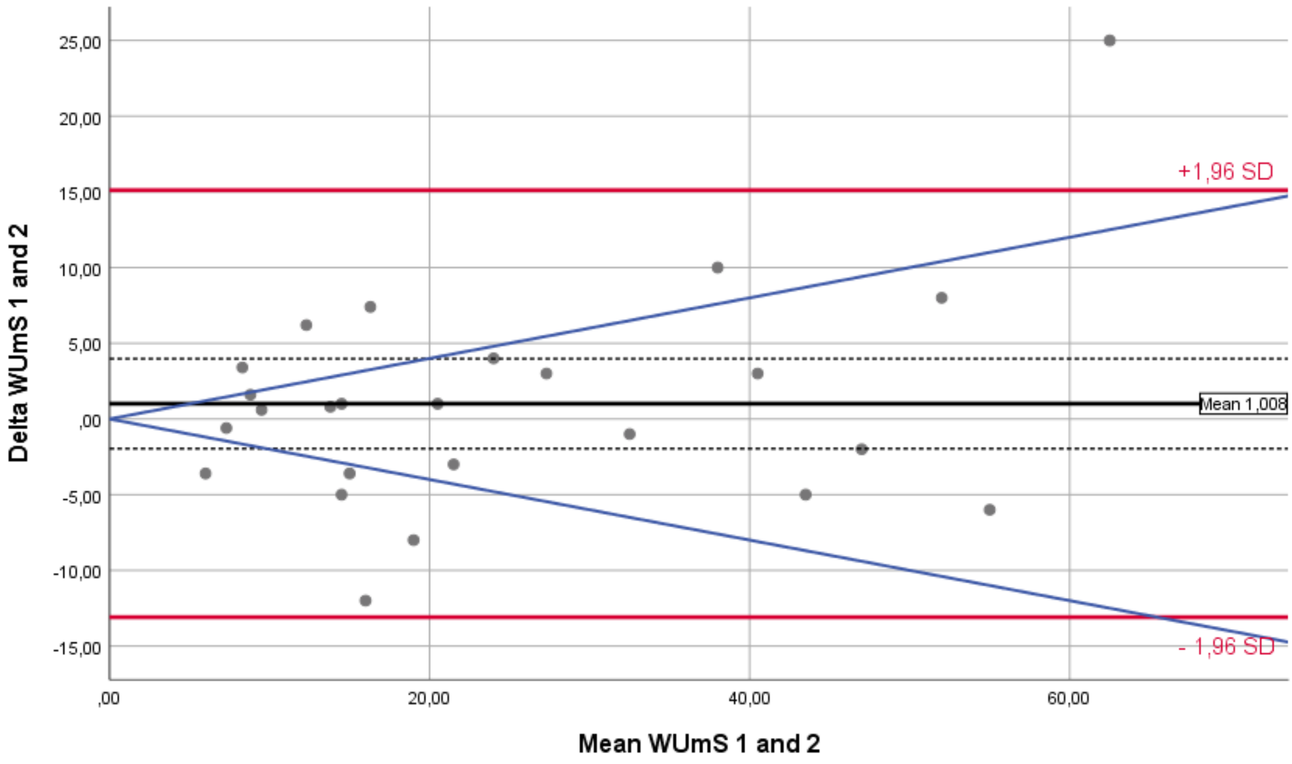

Supporting Information Figure 9:

Bland Altman Plot – Wind Up multiple Stimuli (WUmS) – Waypoints 1 against 2

The solid black lines in the Bland-Altman-Plots represent the mean of the differences.
The confidence intervals for the means of differences are depicted as dashed black lines.
The red upper (lower) lines show the upper (lower) limits of agreement equal to mean ± 1.96 SD.
The blue lines represent a margin of ±20% around the means of measurements from each modality and serve as a possible indicator of clinical relevance.

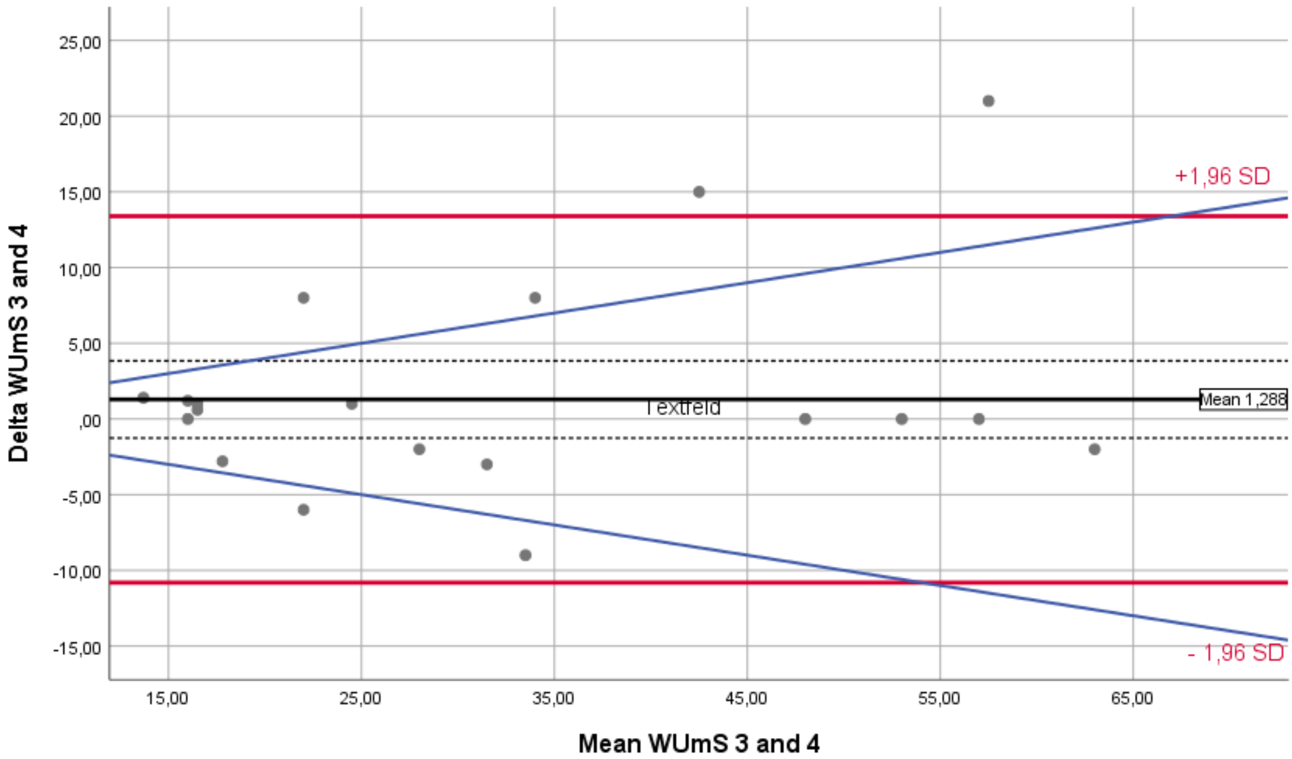

Supporting Information Figure 10:

Bland Altman Plot – Wind Up multiple Stimuli (WUmS) – Waypoints 3 against 4

The solid black lines in the Bland-Altman-Plots represent the mean of the differences.
The confidence intervals for the means of differences are depicted as dashed black lines.
The red upper (lower) lines show the upper (lower) limits of agreement equal to mean ± 1.96 SD.
The blue lines represent a margin of ±20% around the means of measurements from each modality and serve as a possible indicator of clinical relevance.

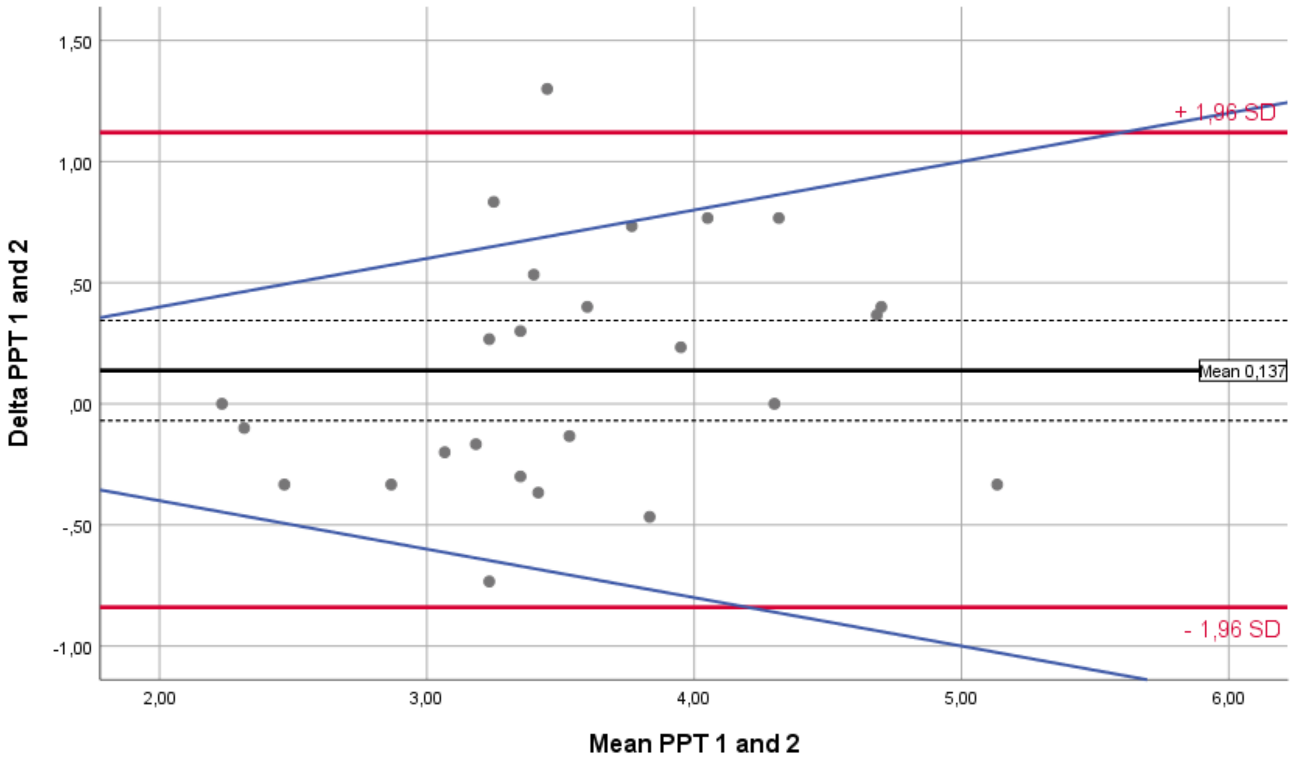

Supporting Information Figure 11:

Bland Altman Plot – Pressure Pain Threshold (PPT) – Waypoints 1 against 2

The solid black lines in the Bland-Altman-Plots represent the mean of the differences.
The confidence intervals for the means of differences are depicted as dashed black lines.
The red upper (lower) lines show the upper (lower) limits of agreement equal to mean ± 1.96 SD.
The blue lines represent a margin of ±20% around the means of measurements from each modality and serve as a possible indicator of clinical relevance.

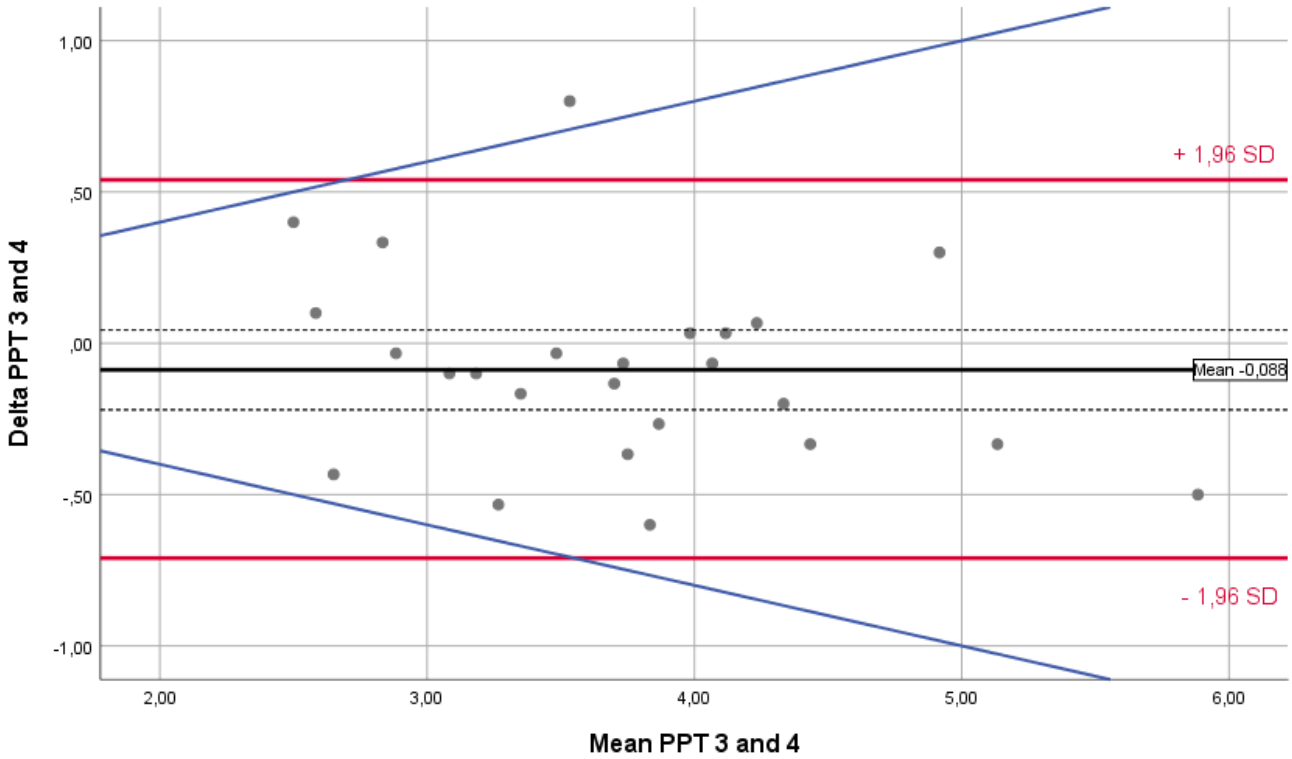

Supporting Information Figure 12:

Bland Altman Plot – Pressure Pain Threshold (PPT) – Waypoints 3 against 4

The solid black lines in the Bland-Altman-Plots represent the mean of the differences.
The confidence intervals for the means of differences are depicted as dashed black lines.
The red upper (lower) lines show the upper (lower) limits of agreement equal to mean ± 1.96 SD.
The blue lines represent a margin of ±20% around the means of measurements from each modality and serve as a possible indicator of clinical relevance.

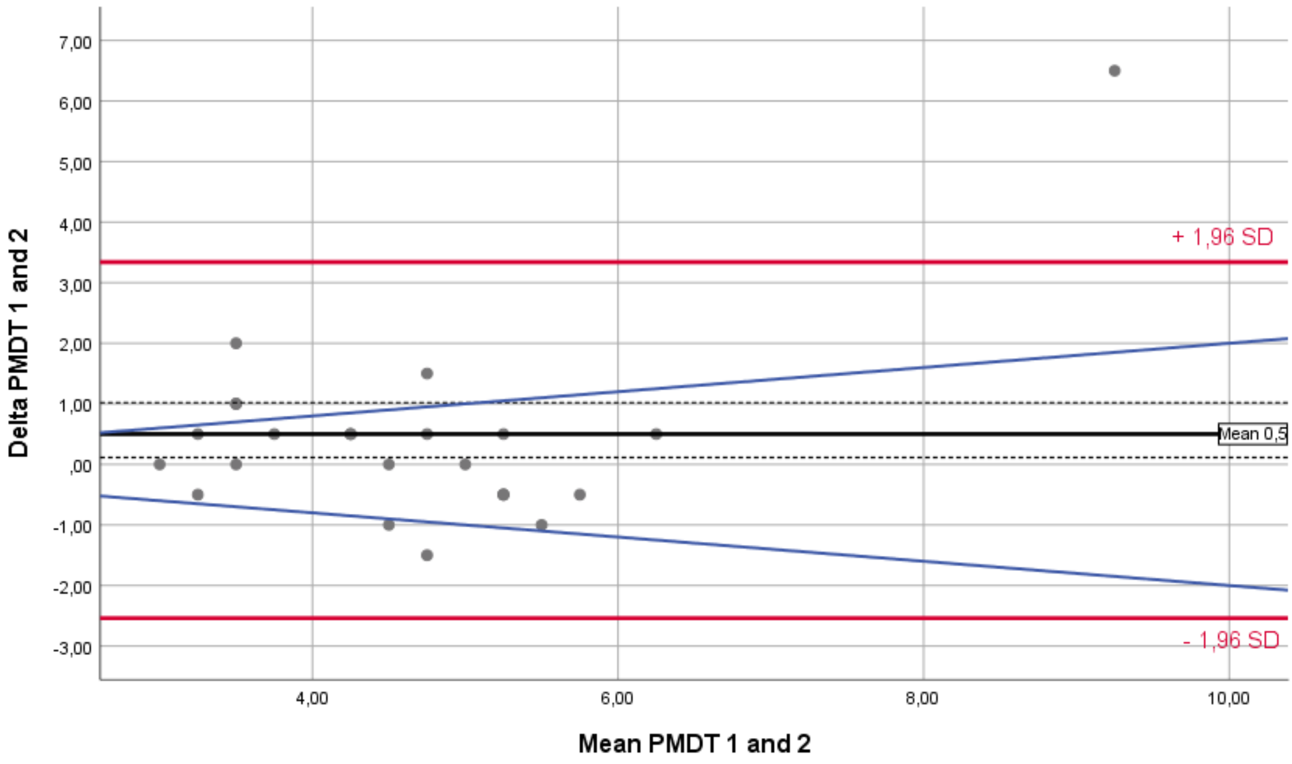

Supporting Information Figure 13:

Bland Altman Plot – Pain Matcher Dectection Threshold (PMDT) – Waypoints 1 against 2

The solid black lines in the Bland-Altman-Plots represent the mean of the differences.
The confidence intervals for the means of differences are depicted as dashed black lines.
The red upper (lower) lines show the upper (lower) limits of agreement equal to mean ± 1.96 SD.
The blue lines represent a margin of ±20% around the means of measurements from each modality and serve as a possible indicator of clinical relevance.

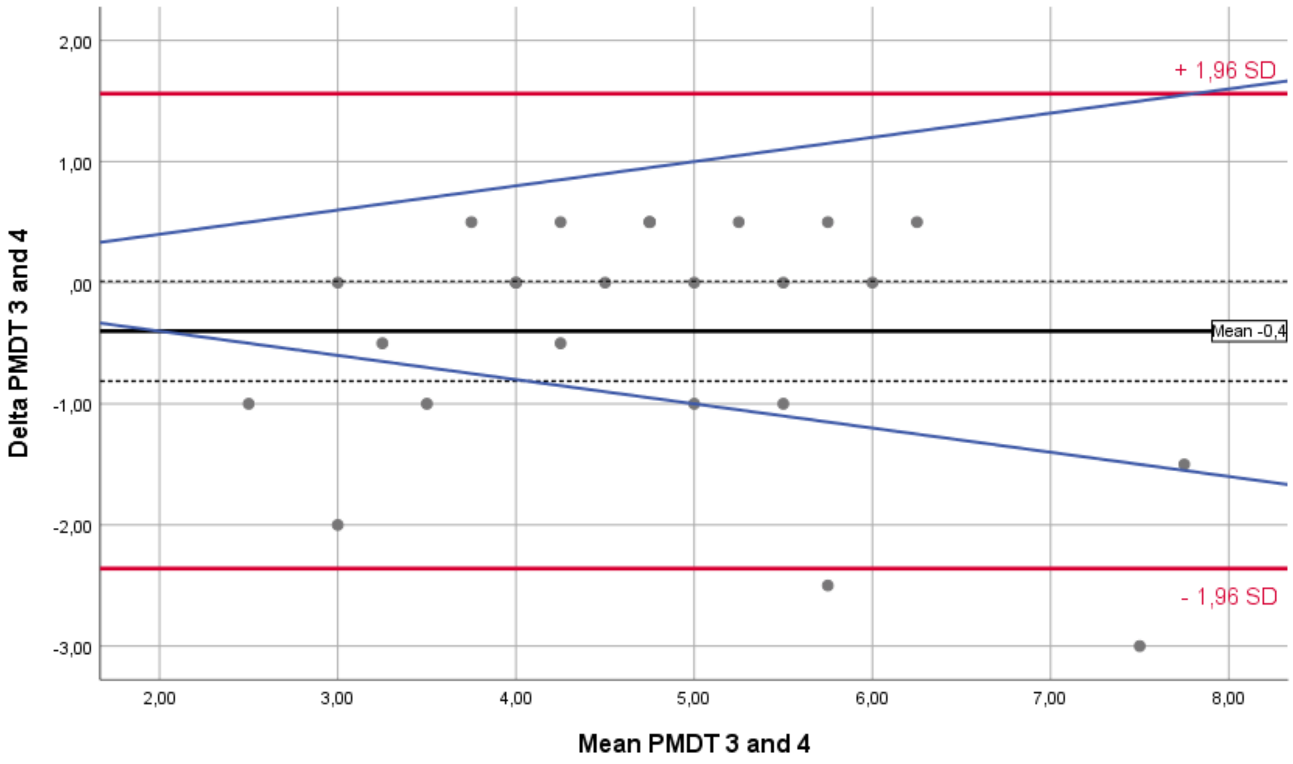

Supporting Information Figure 14:

Bland Altman Plot – Pain Matcher Dectection Threshold (PMDT) – Waypoints 3 against 4

The solid black lines in the Bland-Altman-Plots represent the mean of the differences.
The confidence intervals for the means of differences are depicted as dashed black lines.
The red upper (lower) lines show the upper (lower) limits of agreement equal to mean ± 1.96 SD.
The blue lines represent a margin of ±20% around the means of measurements from each modality and serve as a possible indicator of clinical relevance.

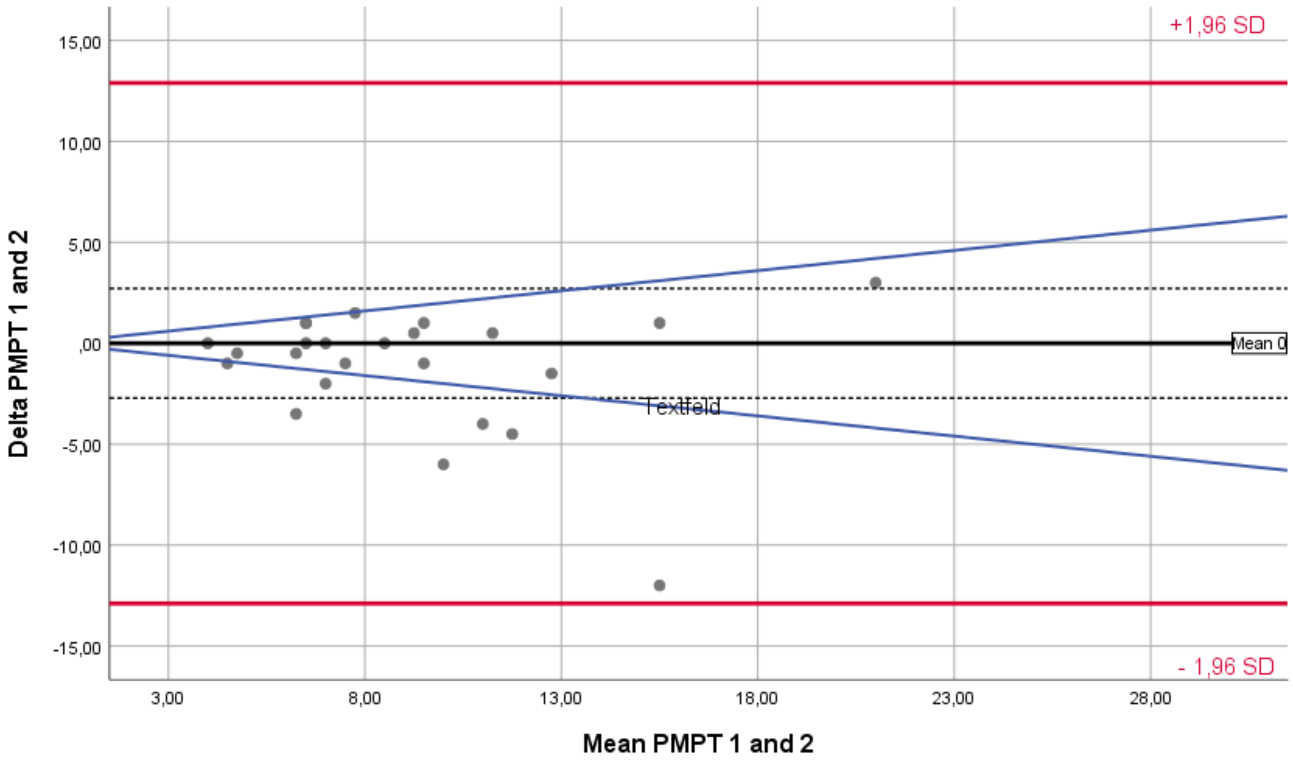

Supporting Information Figure 15:

Bland Altman Plot – Pain Matcher Pain Threshold (PMPT) – Waypoints 1 against 2

The solid black lines in the Bland-Altman-Plots represent the mean of the differences.
The confidence intervals for the means of differences are depicted as dashed black lines.
The red upper (lower) lines show the upper (lower) limits of agreement equal to mean ± 1.96 SD.
The blue lines represent a margin of ±20% around the means of measurements from each modality and serve as a possible indicator of clinical relevance.

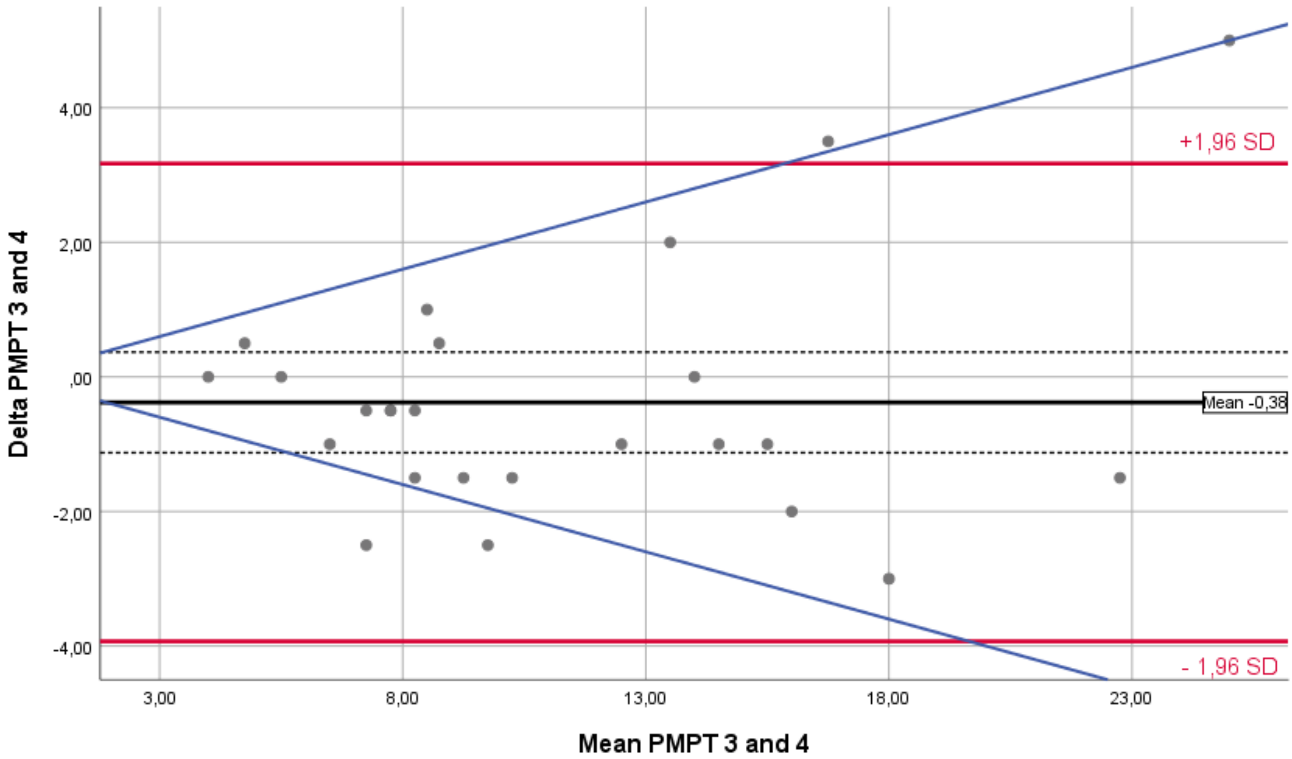

Supporting Information Figure 16:

Bland Altman Plot – Pain Matcher Pain Threshold (PMPT) – Waypoints 3 against 4

The solid black lines in the Bland-Altman-Plots represent the mean of the differences.
The confidence intervals for the means of differences are depicted as dashed black lines.
The red upper (lower) lines show the upper (lower) limits of agreement equal to mean ± 1.96 SD.
The blue lines represent a margin of ±20% around the means of measurements from each modality and serve as a possible indicator of clinical relevance.

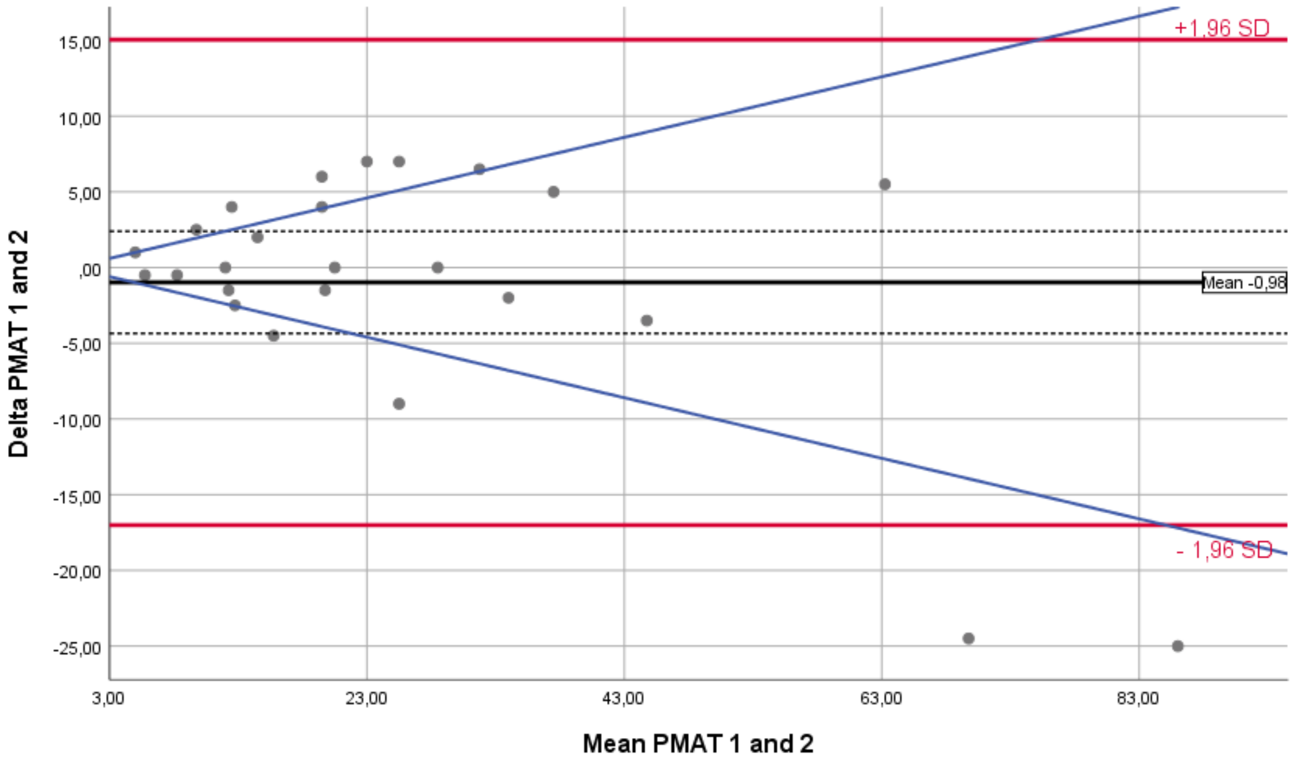

Supporting Information Figure 17:

Bland Altman Plot – Pain Matcher Abort Threshold (PMAT) – Waypoints 1 against 2

The solid black lines in the Bland-Altman-Plots represent the mean of the differences.
The confidence intervals for the means of differences are depicted as dashed black lines.
The red upper (lower) lines show the upper (lower) limits of agreement equal to mean ± 1.96 SD.
The blue lines represent a margin of ±20% around the means of measurements from each modality and serve as a possible indicator of clinical relevance.

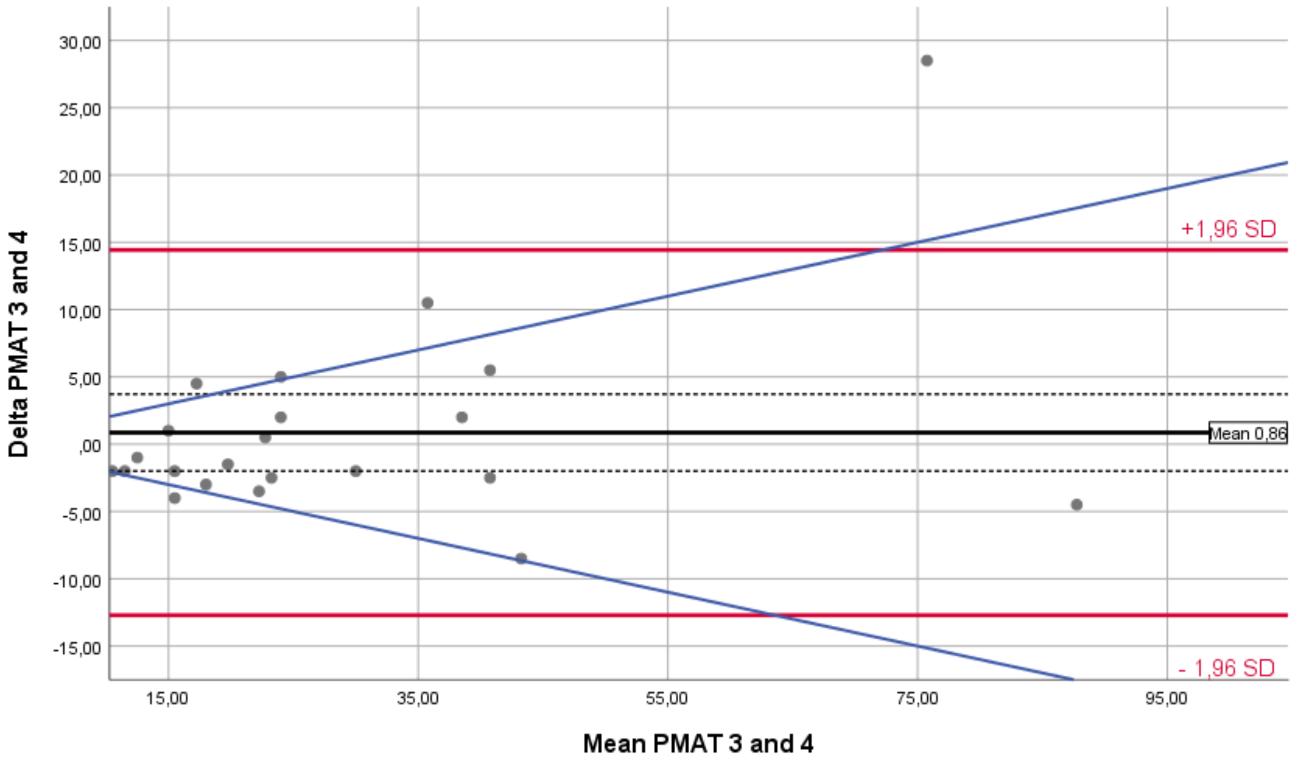

Supporting Information Figure 18:

Bland Altman Plot – Pain Matcher Abort Threshold (PMAT) – Waypoints 3 against 4

The solid black lines in the Bland-Altman-Plots represent the mean of the differences.
The confidence intervals for the means of differences are depicted as dashed black lines.
The red upper (lower) lines show the upper (lower) limits of agreement equal to mean ± 1.96 SD.
The blue lines represent a margin of ±20% around the means of measurements from each modality and serve as a possible indicator of clinical relevance.
